# Microvascular endothelial cells-derived microvesicles imply in ischemic stroke by modulating astrocyte and blood brain barrier function and cerebral blood flow

**DOI:** 10.1186/s13041-016-0243-1

**Published:** 2016-06-07

**Authors:** Qunwen Pan, Caixia He, Hua Liu, Xiaorong Liao, Bingyan Dai, Yanfang Chen, Yi Yang, Bin Zhao, Ji Bihl, Xiaotang Ma

**Affiliations:** Guangdong Key Laboratory of Age-Related Cardiac and Cerebral Diseases, Institute of Neurology, Affiliated Hospital of Guangdong Medical University, Zhanjiang, 524001 China; Guangdong Medical University, Zhanjiang, 524001 China; College of Health Science, Wuhan Sports University, Wuhan, Hubei 430000 China; Department of Pharmacology and Toxicology, Wright State University, Dayton, OH 45435 USA; Department of Neurology, Boonshoft School of Medicine, Wright State University, Dayton, OH 45435 USA

**Keywords:** Endothelial cells, Microvesicles, Astrocytes, Blood brain barrier, Cerebral blood flow, Gene expression, Cerebral ischemia

## Abstract

**Background:**

Endothelial cell (EC) released microvesicles (EMVs) can affect various target cells by transferring carried genetic information. Astrocytes are the main components of the blood brain barrier (BBB) structure in the brain and participate in regulating BBB integrity and blood flow. The interactions between ECs and astrocytes are essential for BBB integrity in homeostasis and pathological conditions. Here, we studied the effects of human brain microvascular ECs released EMVs on astrocyte functions. Additionally, we investigated the effects of EMVs treated astrocytes on regulating BBB function and cerebral ischemic damage.

**Results:**

EMVs prepared from ECs cultured in normal condition (n-EMVs) or oxygen and glucose deprivation (OGD-EMVs) condition had diverse effects on astrocytes. The n-EMVs promoted, while the OGD-EMVs inhibited the proliferation of astrocytes via regulating PI3K/Akt pathway. Glial fibrillary acidic protein (GFAP) expression (marker of astrocyte activation) was up-regulated by n-EMVs, while down-regulated by OGD-EMVs. Meanwhile, n-EMVs inhibited but OGD-EMVs promoted the apoptosis of astrocytes accompanied by up/down-regulating the expression of Caspase-9 and Bcl-2. In the BBB model of ECs-astrocytes co-culture, the n-EMVs, conversely to OGD-EMVs, decreased the permeability of BBB accompanied with up-regulation of zonula occudens-1(ZO-1) and Claudin-5. In a transient cerebral ischemia mouse model, n-EMVs ameliorated, while OGD-EMVs aggravated, BBB disruption, local cerebral blood flow (CBF) reduction, infarct volume and neurological deficit score.

**Conclusions:**

Our data suggest that EMVs diversely modulate astrocyte functions, BBB integrity and CBF, and could serve as a novel therapeutic target for ischemic stroke.

**Electronic supplementary material:**

The online version of this article (doi:10.1186/s13041-016-0243-1) contains supplementary material, which is available to authorized users.

## Background

Ischemic stroke (IS) is a main subtype of stroke causing severe long-term disability or death. Previous studies have suggested that blood brain barrier (BBB) disruption is implicated in the onset and progression of IS [1, 2]. Thus, protection and maintaining BBB functions and its integrity should be important in alleviating brain damage after IS. However, the mechanisms regulating BBB functions are not fully understood and so far, no effective strategy is available for the management of IS induced BBB disruption [3, 4].

Endothelial cells (ECs) and astrocytes are the main components of BBB. Endothelial cells play an important role in BBB function by developing a highly selective barrier. The tight junction proteins such as claudin-5, occludin, and zonula occludens-1 (ZO-1) existing in endothelial cells are the most important proteins modulating the integrity of BBB [5, 6]. Astrocytes also play a pivotal role in maintaining the integrity of BBB via end-feet mediated contact-dependent mechanisms, releasing of trophic factors and promotion of tight junction formation [7–9]. Endothelial-astrocyte interactions and signaling could be essential for BBB integrity and homeostasis in both physiological and pathological conditions [10]. Soluble factors such as TGFβ, GDNF, bFGF, IL-6 and steroids secreted by astrocytes can affect EC permeability [11]. ECs have a reciprocal inductive influence on astrocytes. Brain ECs have been showed to have a trophic influence on astrocytes by secreting PDGF, CNTF, IGF-1 and FGF [12]. Additionally, a leukaemia inhibitory factor released by ECs of the optic nerve has been shown to induce astrocytic differentiation [13]. However, the underlying mechanisms of endothelial-astrocyte interactions are not fully understood.

Microvesicles(MVs) are submicron membrane vesicles released by various cell types in response to different stimuli and deliver proteins and gene messages such as mRNA and microRNA (miRNA) to the recipient cells, representing a novel way of cell-to-cell communication [14, 15]. Our previous study has demonstrated that MVs released from EPCs under stress and apoptotic status have distinguished functions [16]. Specifically, EPCs cultured in serum deprivation (SD) medium decreased ROS production and apoptosis and increased eNOS and NO production of injured ECs. While MVs released from EPCs cultured in SD medium containing tumor necrosis factor-a (TNF-a) are functionally converse on EC apoptosis and dysfunction. MVs secreted from ECs (EMVs) have been suggested to affect the functions of various cells, such as T cells, endothelial cells, leukocytes and smooth muscle cells [17–20]. However, the effects of EMVs on astrocyte and BBB functions, and cerebral ischemic injury have not been determined.

In this study, we investigated the effects of EMVs obtained under normal and oxygen and glucose deprivation condition on astrocytes activities including cell proliferation, apoptosis and GFAP expression, and the underlying mechanisms were also detected. The roles of EMVs on BBB disruption in vitro and in vivo as well as on brain ischemic injury were further studied.

## Results

### The characters of EMVs

**Fig. 1:**
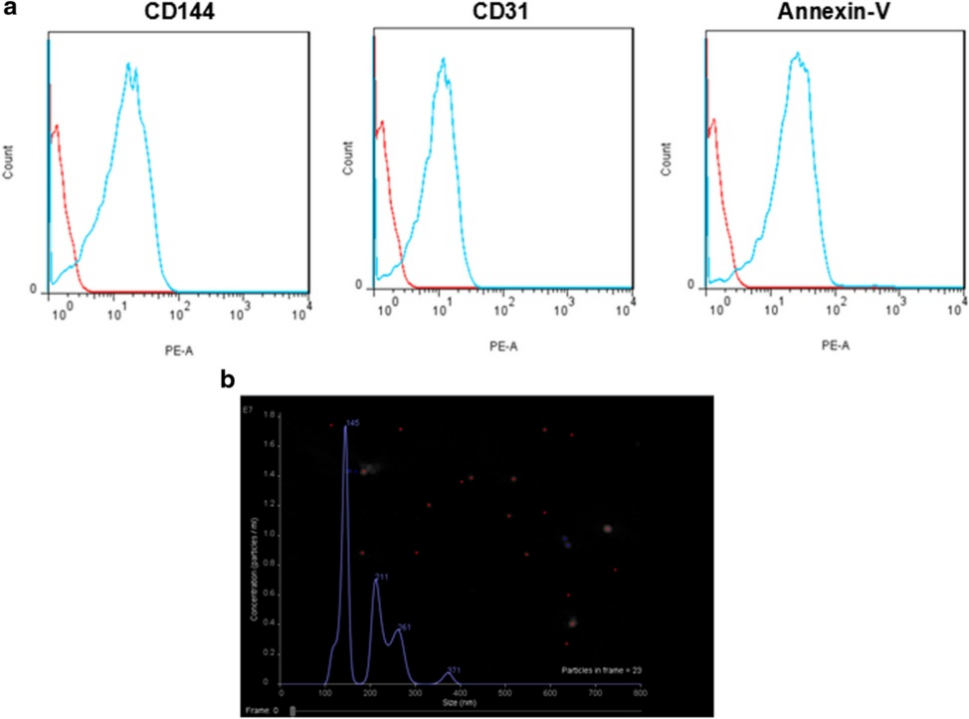
Characterization of EMVs by flow cytometric analysis and NTA. **a** Flow cytometric analysis showing the expression of MV specific marker (Annexin V) and endothelial cell specific markers (CD31 and CD144) in EMVs. **b** NTA analysis confirmed the size distribution of EMVs

Flow cytometric analysis indicated that EMVs positively expressed Annexin V (97 ± 1.5 %), a common marker for MVs detection. In addition, we also found ECs specific marker CD31 (91 ± 1.3 %) and CD144 (92 ± 1.1 %) are highly expressed in EMVs (Fig. 1a). NTA analysis showed that EMVs were in size of 100 nm to 400 nm, and the concentration of EMVs were about 2.5 × 10^10^/30 mL cell culture medium (Fig. 1b).

### EMVs merged with astrocytes after in vitro co-incubation

**Fig. 2:**
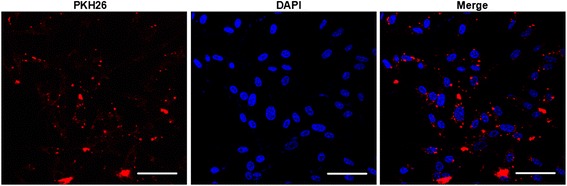
The incorporation of EMVs with astrocytes after coculture (A) Representative images showing that EMVs merged with astrocytes. EMVs were labeled with PKH26 (*red*). Nucleuses were labeled with DAPI (*blue*). Scale bar, 50 μm

After co-incubation of PKH26 labeled EMVs with astrocytes for 24 h, the PKH26 fluorescent was able to be detected in the cytoplasm of astrocytes as revealed by immunofuoresence analysis, suggesting that EMVs merged with astrocytes (Fig. 2).

### The proliferation of astrocytes was differently modulated by n-EMVs and OGD-EMVs via the PI3K pathway

**Fig. 3:**
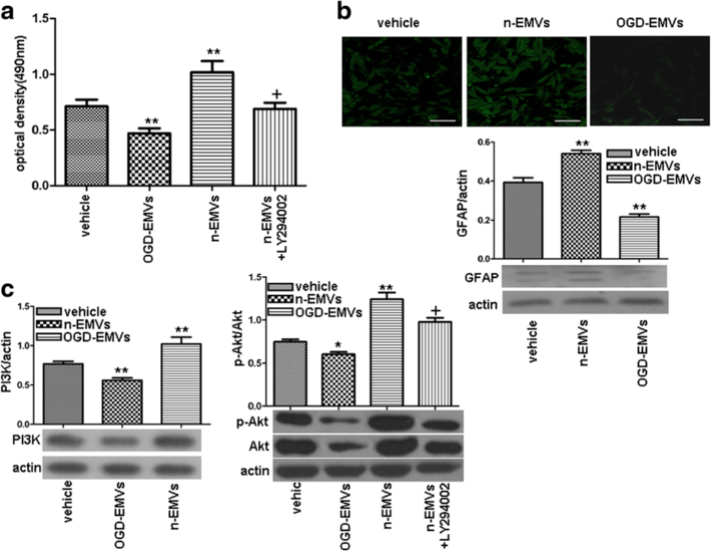
The effects of n-EMVs and OGD-EMVs on astrocyte proliferation and expression of GFAP and PI3K/Akt were opposite. **a** MTT assay of astrocyte proliferation. **b** Representative images and quantitative analysis of GFAP in each group. Scale bar, 50 μm. **c** Expression of PI3K and p-Akt/Akt. (^**^ *p* <0.01,vs. vehicle; ^+^ *p* <0.05,vs. n-EMVs, *n* = 5)

As shown in Fig. 3a–c, n-EMVs markedly increased the proliferation (1.06 ± 0.17 of n-EMVs group vs. 0.75 ± 0.07 of vehicle; *p* <0.01; Fig. 3a) and GFAP expression (0.54 ± 0.03 of n-EMVs group vs. 0.39 ± 0.04 of vehicle; *p* <0.01; Fig. 3b) of astrocytes, paralleled with up-regulation of PI3K and p-Akt/Akt level (vs.vehicle; *p* <0.01; Fig. 3c). However, OGD-EMVs decreased proliferation (0.49 ± 0.05 of OGD-EMVs group vs. 0.75 ± 0.07 of vehicle; *p* <0.01; Fig. 3a) and GFAP expression (0.22 ± 0.026 of n-EMVs group vs. 0.39 ± 0.04 of vehicle; *p* <0.01; Fig. 3b) of astrocytes, when companied with down-regulation of PI3K expression, p-Akt/Akt level (vs.vehicle; *p* <0.05; Fig. 3c) after 3 days incubation.

In addition, preincuabation of astrocytes with PI3K inhibitor (LY294002) abolished the aforementioned effects of n-EMVs (*vs* n-EMVs; *p* <0.05; Fig. 3c), suggesting that the beneficial effects of n-EMVs are mediated by the PI3K pathway.

### N-EMVs decreased whereas OGD-EMVs increased the apoptosis of astrocytes via modulating the expression of cleaved Caspase-9 and Bcl-2

**Fig. 4:**
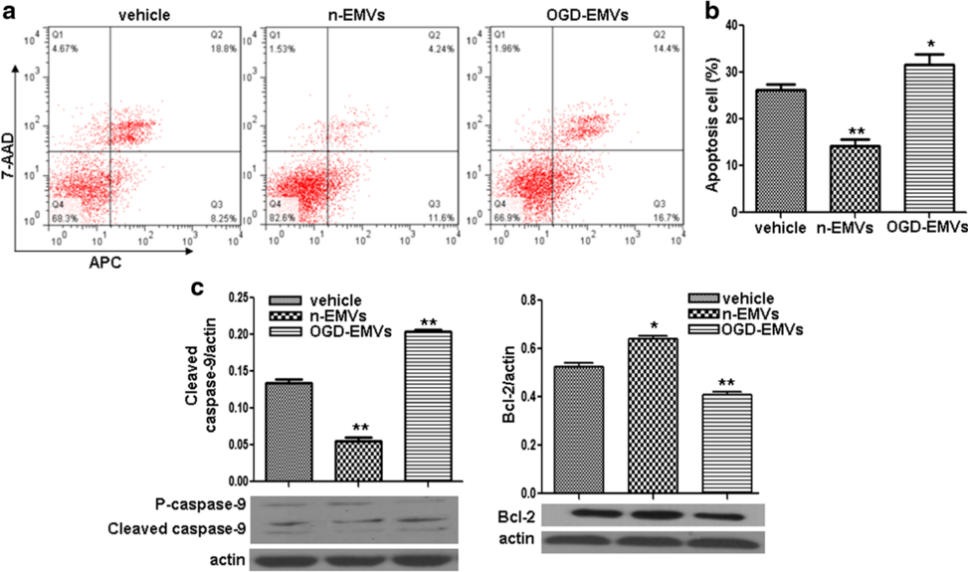
The effects of n-EMVs and OGD-EMVs on astrocyte apoptosis and expression of cleaved Caspase-9 and Bcl-2. **a** Representative flow cytometric analysis of astrocytes apoptosis. **b** Summarized data on the percentage of apoptotic astrocytes in each group. **c** Cleaved Caspase-9 and Bcl-2 expression in astrocytes. (^*^ *p* <0.05,^**^ *p* <0.01,vs. vehicle, *n* = 5)

Annexin V-APC/7-AAD analysis revealed that n-EMVs decreased while OGD-EMVs increased apoptotic rate of astrocytes (vs. vehicle; *p* <0.05 or 0.01; Fig. 4a, b). The efficency of n-EMVs on decreasing astrocytes apoptosis was about 12 %. Whereas, the apoptosis rate of astrocytes was increased by approximately 5 % after co-cultured with OGD-EMVs.

In addition, we monitored the cleaved Caspase-9 and Bcl-2 levels, which are associated with induction of apoptosis, by western blot. Results (Fig. 4c) showed that cleaved Caspase-9 protein expression was significantly increased (vs. vehicle; *p* <0.01) but Bcl-2 protein expression obviously decreased (vs. vehicle; *p* <0.01; Fig. 4c) by OGD-EMVs treatment; whereas, n-EMVs had opposite effects on the expressions of cleaved Caspase-9 and Bcl-2 (vs. vehicle; *p* <0.05 or *p* <0.01; Fig. 4c).

### EMVs pretreatment of astrocytes modulated the permeability of in vitro BBB model and the expression of ZO-1 and claudin-5 in ECs

**Fig. 5:**
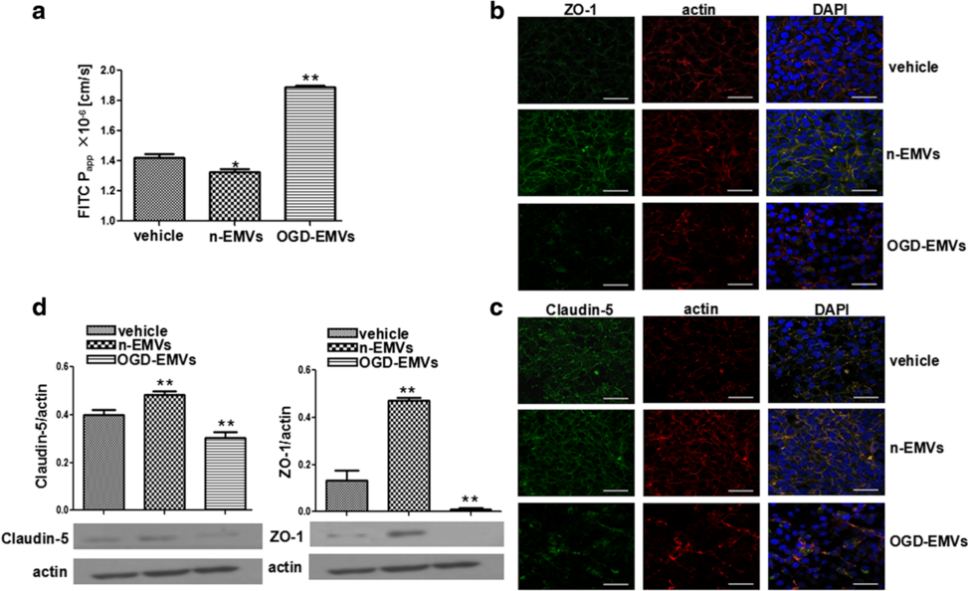
Treatment with n-EMVs and OGD-EMVs reversely influenced the permeability of BBB and ZO-1/Claudin-5 expression. **a** n-EMVs reduced while OGD-EMVs increased the permeability of BBB. **b**, **c** Representative images of ZO-1 (**b**) and Claudin-5 (**c**) staining on HBMECs. Scale bar, 50 μm. **d** Western blot analyses of ZO-1 and Claudin-5. (^*^ *p* <0.05, ^**^ *p* <0.01, vs. vehicle, *n* = 5)

As shown in Fig. 5, n-EMVs incubated astrocytes decreased the paracelluar permeability of HBMECs examined by FITC-Dextran Flux (1.3 ± 0.14 × 10^−6^ cm/s of n-EMVs group vs. 1.5 ± 0.14 × 10^−6^ cm/s of vehicle; *p* <0.05; Fig. 5a), accompanied with the up-regulation of zonula occudens-1(ZO-1) (vs. vehicle; *p* <0.01; Fig. 5b, d) and Claudin-5 expression at protein levels (vs. vehicle; *p* <0.01; Fig. 5c, d). While OGD-EMVs incubated astrocytes increased the paracelluar permeability of HBMECs (1.9 ± 0.17 × 10^−6^ cm/s of OGD-EMVs group vs. 1.5 ± 0.14 × 10^−6^ cm/s of vehicle; *p* <0.01; Fig. 5a) accompanied with the decrease in expression of ZO-1 (vs. vehicle; *p* <0.01; Fig. 5b, d) and Claudin-5(vs. vehicle; *p* <0.01; Fig. 5c, d), resulting in a discontinuous distribution of ZO-1 (b) and Claudin-5 (c) on the membrane of HBMECs.

### EMVs merged with astrocytes in brain tissue

**Fig. 6:**
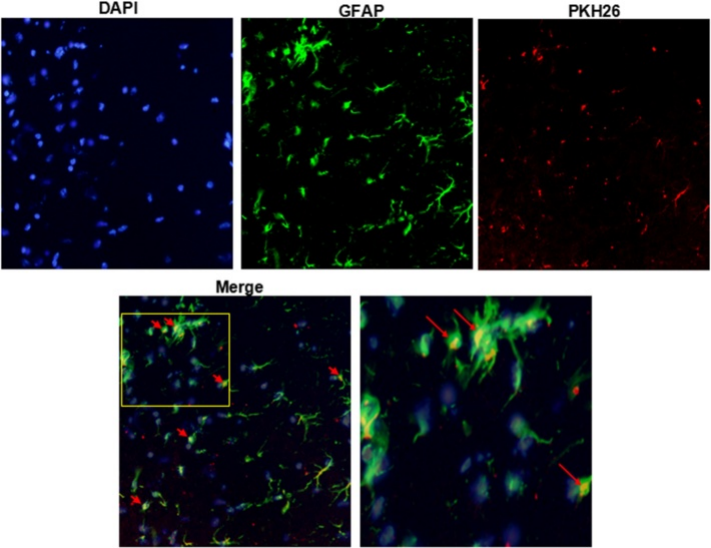
Representative images showing that injected EMVs merged with astrocytes in the peri-infarct area. EMVs labeled with PKH26 (*red*), and astrocytes labeled with GFAP (*green*). Nucleuses stained with DAPI (*blue*). Magnification, 400×

The PKH26 labeled EMVs were injected into mice for 24 h. Astrocytes in brain tissue samples were shown by GFAP staining. The PKH26 fluorescent was able to be detected in the cytoplasm of the astrocytes in mouse brains, demonstrating that EMVs could merge with astrocytes in brain tissue (Fig. 6).

### N-EMVs reduced while OGD-EMVs increased Evans blue extravasation of BBB in tMCAO mice

**Fig. 7:**
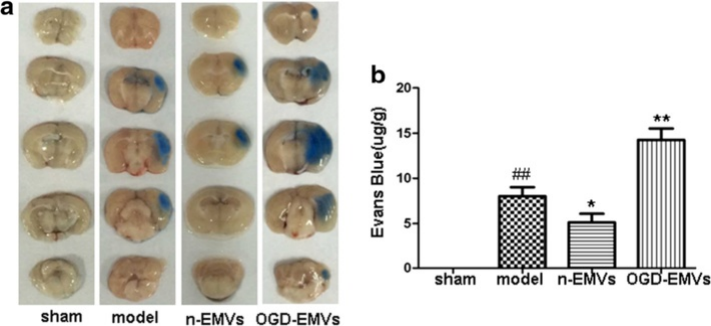
N-EMVs reduced while OGD-EMVs promoted evans blue extravasation in tMCAO mice. **a** The representative pictures of Evans blue extravasation in ischemic right brains of various groups. **b** The quantitative analysis of Evans blue leakage. (^*^ *p* <0.05, ^**^ *p* <0.01,vs. model group, *n* = 5)

BBB permeability was tested by Evans blue dye extravasation at the whole brain of mice in various groups. As shown in Fig. 7a–b, Evans blue dye extravasation in ischemia/reperfusion (model) group was markedly increased after 48 h reperfusion (8.4 ± 0.51 μg/g mouse brain) compared with the sham group (vs. sham; *p* <0.01; Fig. 7b), which was attenuated significantly by n-EMVs treatment (4.8 ± 0.31 μg/g mouse brain) (vs. model; *p* <0.05; Fig. 7b). However, the extravasated evans blue in brain were significantly increased in OGD-EMVs transfusion group (14.7 ± 0.81 μg/g mouse brain) compared to the model group (vs. model; *p* <0.01; Fig. 7b).

### Infusion of n-EMVs decreased while OGD-EMVs aggravated CBF reduction, cerebral injury and neurological deficits of tMCAO mice

**Fig. 8:**
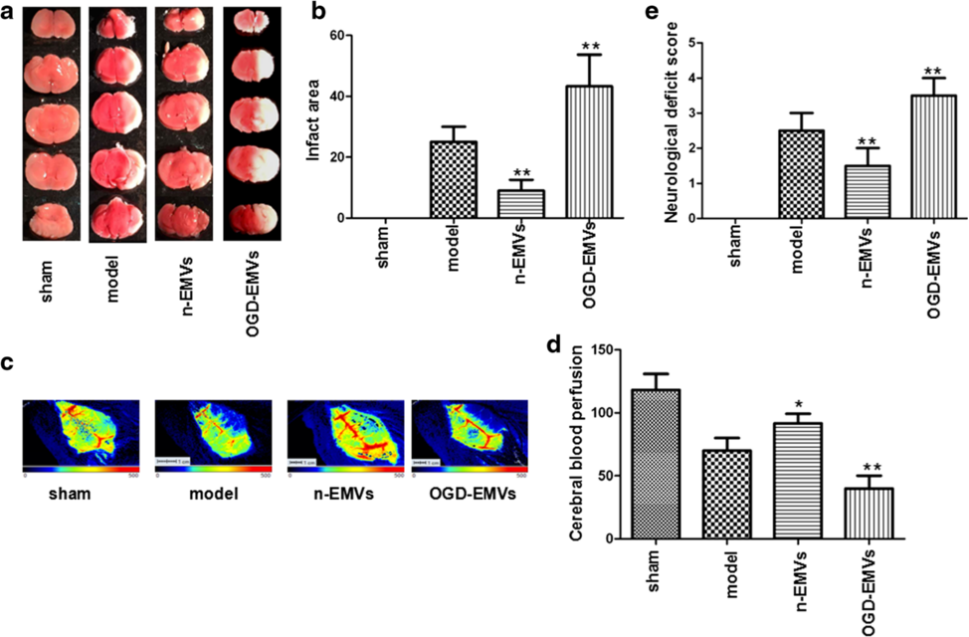
Effects of EMVs on infract area, neurological deficits score and CBF. **a**–**b** Representative brain TTC staining and quantitative analysis of infarct size in different groups. **c**–**d** The representative images and analysis of CBF in different groups. *Blue* to *red* represent low to high perfusion. **e** Neurological deficits score in different groups. (^*^ *P* <0.05, ^**^ *p* <0.01 vs. model group, *n* = 5)

As shown in Fig. 8a–e, n-EMVs significantly improved CBF (*p* <0.05; Fig. 8c–d), and reduced infarct volume compared to model group (vs. model; *p* <0.01; Fig. 8a–b) 48 h after tMCAO following EMVs transfusion. Meanwhile, n-EMVs also reduced the neurological deficit score (vs. model; *p* <0.01; Fig. 8e) at 48 h after tMCAO. On the contrary, OGD-EMVs aggravated the cerebral injury by decreasing CBF (vs. model; *p* <0.01; Fig. 8c–d) and increasing infarct volume (vs. model; *p* <0.01; Fig. 8a–b) 48 h after tMCAO. In addition, OGD-EMVs also promoted neurologic deficit at 48 h after MCAO following EMVs transfusion (vs. model; *p* <0.01; Fig. 8e).

## Discussion

There are three major findings in this study. Firstly, we demonstrated that n-EMVs released from normal conditions increased cell proliferation, GFAP expression and decreased cell apoptosis, while OGD-EMVs obtained from oxygen and glucose deprivation condition had opposite effects. Moreover, we revealed that the effects of EMVs were linked with PI3K/Akt and Bcl-2 and Caspase-9 signaling pathways. Secondly, n-EMVs and OGD-EMVs played different roles in regulating BBB function via modulating the expression of Claudin-5 and ZO-1 in ECs. Finally, in the mouse tMACO model, we demonstrated that n-EMVs preserved BBB function and CBF, decreased the infarct volume and neurological deficits, whereas OGD-EMVs had opposite effects.

It’s well known that astrocytes perform a very important role in the physiology and pathology of the brain, such as neuronal survival and cerebral repair [21, 22]. In response to ischemic brain damage, astrocytes are activated and form a glial scar, which protects the healthy tissue from cascading uncontrolled tissue damage. Meanwhile, activated astrocytes produce neurotrophic factors and take up excessive glutamine, which help to protect neurons and reduce neuronal injury [23, 24]. Thus, it is important to identify the relevant glial mechanisms in ischemic stroke.

MVs are submicron membrane fragments released from virtually all cell types upon activation, apoptosis and stress, which have been documented as potential biomarkers and indicators for diseases [25, 26]. Of note, several studies have demonstrated that MVs released from different stimuli exert different functions to recipient cells [27, 28]. Herein, we verified that EMVs fused with astrocytes and regulated the proliferation, apoptosis and GFAP expression of astrocytes. Moreover, we found that the n- EMVs promoted while OGD- EMVs inhibited the proliferation and the GFAP expression of astrocytes. GFAP has been considered as a specific marker of astrocyte activation [29, 30]. These indicated that n-EMVs may help maintaining astrocyte function and internal environment homeostasis of the brain, while OGD-EMVs showed opposite effects on astroctyes, which may participate in the pathological processes of ischemic damage. In fact, activation of astrocytes may play controversial function roles in cerebral damage. For example, astrocyte over-activation might inhibit axonal regeneration by elevating various inhibitory molecules, such as chondroitin sulface proteoglycans [31].

PI3K/Akt pathway participates in regulating a wide range of cellular processes including proliferation, differentiation, angiogenesis and survival [30, 32]. There is evidence showing that the PI3K/Akt pathway regulates the proliferation of astrocytes [31, 33]. Caspase-9 participates in the initiation and execution of cell apoptosis [34]. Bcl-2, a nuclear factor-kβ, possesses anti-apoptotic effects by maintaining mitochondrial homeostasis [35]. In coculture experiments, we found that n-EMVs promoted astrocyte proliferation while OGD-EMVs produced opposite effects on astrocytes, which were abrogated by PI3K inhibitor LY294002. In addition, our results demonstrated that n-EMVs reduced astrocyte apoptosis paralleled with decreased protein expression of cleaved Caspase-9 and increased Bcl-2 expression, while OGD-EMVs promoted astrocyte apoptosis accompanied with up-regulation of cleaved Caspase-9 and down-regulation of Bcl-2. These data suggested that regulation of the PI3K/Akt pathway and Caspase-9/Bcl-2 pathways could be the underlying mechanisms of EMVs in control of astrocyte proliferation and apoptosis. Of note, our data revealed the counteractive functions of n-EMVs and OGD-EMVs. Similar situations have been previously reported by others [36] and us [16]. It could be related to their differences in their carried messages as suggested by these reports [16, 36]. Nevertheless, the differences of the cargo contents existed between n-EMVs and OGD-EMVs need further exploration.

Astrocytes are the main cell components of BBB structure and participate in maintaining BBB integrity and protecting the brain from various damage attacks [37, 38]. In order to confirm the role of n-EMVs and OGD-EMVs in BBB function, we constructed transwell-based BBB in vitro model. In the in vitro BBB model, we found that n-EMV treatment improved the BBB function by increasing tight junction proteins Claudin-5 and ZO-1 expression in HBMECs, which are the most important components for endothelial cell barrier integrity [39, 40]. However, OGD- EMV treatment increased the permeability of BBB and reduced the tight junction proteins Claudin-5 and ZO-1 expression in ECs.

We advanced our in vitro studies on EMVs to in vivo study in the mouse tMCAO model. BBB disruption is involved in various brain injuries, such as intracranial hemorrhage, ischemic stroke and cerebral trauma [41–43]. Moreover, BBB disruption increases cerebrovascular permeability, allowing the entrance of inflammatory factors and leukocytes into the brain parenchyma which in turn contributed to secondary brain injury [44, 45]. In this study, we generated BBB in vivo disruption from focal transient ischemic stroke, and the degree of BBB disruption is assessed by evens blue extravasation. Our results showed that n-EMVs decreased while OGD-EMVs increased the evens blue exravasation after the focal transient ischemic stroke induced BBB disruption, which consistent with the effects of EMVs on in vitro BBB model. These results suggested that EMVs could regulate astrocytes activities and consequently modulate BBB functions. And EMVs released under different conditions may exert opposite roles.

Ischemic brain damage is an extremely complex multi-factors pathological process accompanied with structural and functional changes of BBB [46]. Many factors, such as plasmin, gelatinases, free radicals, inflammatory factor, vasoactive substances, neuroglia and so on, are involved in the control of BBB permeability, implying in ischemic cerebral injury [47, 48]. In this study, we further determined the effects of EMVs on ischemic injury by intravenous infusion of n-EMVs or OGD- EMVs into C57 mice after tMCAO surgery. We found that n-EMVs improved CBF and protected brain from ischemic injury. On the contrary, OGD-EMVs showed deleterious effects (increasing the infarct volume and neurological deficit score, decreasing CBF). CBF is essential for brain oxygen supply, and brain ischemic injury is closely related to reductions in CBF [49]. Decreased CBF is the most important indicator of ischemic stroke [50]. Astrocytes have been shown to be involved in the regulation of CBF by mediating vasodilation [51]. Thus, the beneficial effects of n-EMVs on astrocyte function may contribute to the CBF preservation and subsequently ameliorated ischemic injury. While the deleterious effects of OGD-EMVs on astrocytes may compromising the CBF. Collectively with our in vitro findings, we suppose that BBB integrity and function and CBF regulation might be one of the mechanisms underlying the effects of n-EMVs and OGD-EMVs on cerebral ischemic injury [52]. Nevertheless, more studies on the dosage and timing of their administration, and detailed mechanisms are needed.

## Conclusion

In conclusion, the present study demonstrates that n-EMVs and OGD-EMVs have opposite effects on regulating astrocyte activities and BBB integrity via regulating the PI3K/AKT and Caspase-9/Bcl-2 signal pathways and that n-EMVs and OGD-EMVs have differently impacts CBF and cerebral ischemic damage, which could offer novel therapeutic strategies for ischemic stroke and BBB disruption related diseases.

## Methods

### Animals

Adult male C57BL6/J mice (6–8 weeks, 20–24 g) were used for the study. The animal protocol was approved by the Ethics Committee of the First Affiliated Hospital of Guangdong Medical College in accordance with the guidelines of the National Institutes of Health (NIH) on the care and use of animals.

### Cell culture

Human brain microvescular ECs were obtained from Shanghai Bioleaf Biotech Co. Ltd, and human astrocytes were purchased from Guangzhou Jennio Biotech Co., Ltd. The cells were cultured on 100-mm cell culture dishes in Dulbecco’s Modified Eagle Medium (DMEM) supplemented with 10 % fetal bovine serum (FBS, GiBCO) containing 100 U/ml of penicillin G and 100 mg/ml of streptomycin, in a 37 °C incubator with humidified atmosphere of 5 % CO_2_/95 % air.

### Preparation and characterization of EMVs

EMVs were generated from human brain microvescular ECs under normal condition culture medium (n-EMVs) or oxygen and glucose deprivation culture medium (OGD-EMVs). In brief, ECs were cultured in 100-mm cell culture dishes. When cells grow to 80 % confluence, cells were washed with PBS and cultured in fresh growth culture medium or glucose deprivation medium under oxygen deprivation condition (1 % O_2_) for 24 h. Then the cell medium was collected and centrifuged at 300 g for 15 min, and followed by 2000 g × 30 min to remove cells and cell debris. The cell-free culture medium was centrifuged at 20,000 g for 2 h to pellet EMVs [53, 54]. The protein concentration of EMVs was quantified by the bradford method (beyotime, china). A concentration of 50 μg/mL EMVs was used for coculture experiments. The size distribution of the EMVs was confirmed by Nanoparticle Tracking Analysis (NTA). EMVs were also analyzed by flow cytometry as previously reported [55]. In brief, EMVs were resuspended and respectively incubated with Alexa-488-labeled Annexin V, PE-conjuated CD144 and CD31 for 15 mins at 4 °C in the dark. Nonspecific isotype antibodies served as negative controls. All antibodies were purchased from eBioscience (San Diego, CA).

### Coculture assay of EMVs with astrocytes

EMVs were labeled with PKH26 (261026 M; Sigma-Aldrich, St. Louis, MO) according to the manufacturer’s protocol with some modifications [56]. In brief, EMVs were labeled with 2 μM PKH26 (261026 M; Sigma-Aldrich, St. Louis, MO) at room temperature (RT) for 5 min [57]. An equal volume of 1 % bovine serum albumin (BSA) was added to stop staining. EMVs were then ultracentrifuged and resuspended with culture medium. The PKH26 labeled EMVs were added to astrocytes seeded in glass bottom plate for 24 h incubation (37 °C, 5 % CO_2_). Cell nuclei were then stained with DPAI (1 μg/ml; Wako Pure Chemical Industries Ltd). The interaction between EMVs and astrocytes was examined under fluorescence microscope (Laica, TCS SP5II, Germany).

### Cell proliferation assay

Proliferative capability of astrocytes was tested by MTT 3-[4,5-dimethylthiazyol-2yl]-2,5-diphenyltetrazolium bromide) (Sigma, 5 mg/ml) assay [58]. Astrocytes were seeded at 1.5 × 10^3^/96-well plate and cultured in 100 μL DMEM (supplemented with 10 % FBS) with n-EMVs, OGD-EMVs or vehicle (PBS). MTT solution (20 μL) was added and incubated with cells for 4 h at 37 °C, then 150 μL DMSO was added to each wells and incubated with the cells for 20 min at 37 °C. The optical density (OD) value of cells was read at 490 nm in a microplate reader (BioTek, USA). Measurement was carried out on day 3 after the incubation. The experiment was repeated three times. Results were calculated from the values gained in three independent experiments. For pathway blocking experiments, cells were pre-incubated with LY294002 (20 μM) for 2 h.

### Annexin V-APC/7-AAD staining analysis of cell apoptosis

Cell apoptosis was analyzed by annexin V-APC/7-AAD staining as previously described [58]. In brief, the serum deprivation medium was used for inducing apoptosis [59]. Cells of each group seeded on sterile cover glasses were placed in 6-well plates for culture in DMEM medium supplemented with vehicle (PBS), n-EMVs or OGD-EMVs for 24 h. The apoptosis assay of atrocytes was conducted with using an Annexin V-APC/7-AAD apoptosis detection kit (BD Biosciences). Briefly, cells were washed with PBS, resuspended with 100 μL 1 × annexin-binding buffer, incubated with 5 μL APC-conjugated Annexin V and 5 μL 7-Amino-actinomycin (7-AAD) for 15 min in the dark, then analyzed by flow cytometry. Cells stained with both Annexin V-APC and 7-AAD were considered to be late apoptotic astrocytes, and the cells stained only with Annexin V-APC were considered to be early apoptotic astrocytes. The experiment was repeated three times, and three plates per experiment were analyzed in each group.

### Paracellular permeability assay of in vitro BBB model

Paracellular permeability assay of BBB was conducted as previously described [60]. Briefly, astrocytes were seeded at a density of 5 × 10^4^ cells/well in 1 mL medium under the bottom chamber of 24-well plate. After cells grown to 80 % confluence, PBS, n-EMVs or OGD-EMVs were added to the bottom chamber and co-cultured with astrocytes. After 24 h co-culture, the culture medium will be replaced with fresh medium. Before astrocytes seeded, ECs were seeded at a density of 2 × 10^4^ cells/well in 300 μL medium onto polycarbonate 24-well transwell chambers with a 0.4 mm mean pore size and a 0.3 cm^2^ surface area (Millicell Hanging Cell Culture Inserts, USA) to form EC barrier. Continuous permeability of ECs was detected for 7 days to determine the timing of EC barrier formation. On day 3 (the day before the barrier formation), the transwell chambers were transfered to EMVs treated astrocytes to compose in vitro BBB model. After 24 h ECs-Astrocytes co-culture, flux of FITC-conjugated dextran (FITC-dextran, 10 kDa, Sigma) across HBMEC monolayer was used to measure the paracellular permeability. HBMECs were incubated with FITC-dextran (1 mg/mL) in HBSS buffer for 90 min. Thereafter, relative fluorescence passed through the chamber (in the lower chambers) was determined by using EnSpire Manager (PerkinElmer Company, USA) multimode plate reader at an excitation wavelength of 485 nm and an emission wavelength of 535 nm. Restriction of paracellular transport was determined by analyzing the apparent permeability coefficient (Papp) for FITC-dextran across the cells. Papp was calculated by the following equation

$$ Papp=\frac{dQ}{dt}\cdot \frac{1}{A\cdot C0\cdot 60}\left( cm/s\right) $$

where dQ/dt is the amount of FITC transported per minute (ng/min), A is the surface area of the filter (cm^2^), C_0_ is the initial concentration of FITC(ng/ml) and 60 is the conversion from minutes to seconds [61, 62].

### Immunofluorescence assay for tight junction proteins

Astrocytes were seeded at a density of 2 × 10^5^ cells/well onto a 6-well plate. Then the confluent astrocytes were cultured with PBS, n-EMVs and OGD-EMVs. After 24 h co-culture, culture medium will be replaced with fresh medium and cells will be cultured for another 24 h. Then, the culture medium was harvested and used to culture HBMECs seeded at the bottom of wave plates. Immunofluorescence assay for tight junction proteins of HBMECs was performed as previously described [63]. HBMECs were incubated with fluorescein isothiocyanate (FITC)-conjugated primary antibodies(Claudin-5, 1:50; ZO-1, 1:50) over night at 4 °C Then, cells were washed triple using wash buffer and incubated with dye for F-actin (Rhodamine Phalloidin, 1:1000) for 1 h at room temperature. DAPI (1:1000) was used for staining cellular nuclear. The cells were washed for three times and observed under a fluorescence microscope (Laica, TCS SP5II, Germany).

### Western blot analysis

Astroctyes were harvested after co-cultured with PBS, n-EMVs or OGD-EMVs, HBMECs were harvested for tight junction protein expression detection. Cells were lysed in ice-cold RIPA (Applygen Technologies Company, Beijing) containing protease for western blot analysis, total cell proteins (40 μg) extracted from each group were separated by 12 % SDS-PAGE on tris-glycine gels (Invitrogen) and transferred to polyvinylidene difluoride membranes (Millipore Corp, Bedford, MA). After blocking at room temperature (RT) in TBS (50 mM Tris, 150 mM NaCl, pH 7.6, 5 % fat-free dry milk) for 1 h, the membranes were washed in TBST (0.5 % Tween 20 in TBS) at RT. Primary antibody was added over night at 4 °C. Following extensive washing, membranes were incubated with secondary antibody (1:50,000, EarthO*x*, San Francisco, CA, USA) for 1 h at RT. After washing 3 times for 30 min with TBST, the immunoreactivity was visualized by ECL solution (Amersham, Sweden). Beta actin (1:1000, EarthOx, San Francisco, CA, USA) was used to normalize protein loading. The following primary antibodies were used: GFAP (1:500, Santa, USA), Caspase-9 and Bcl-2(1:1000, CST, USA), PI3 kinase p110a (1:1000, CST, USA), Akt (1:1000, CST, USA), p-Akt (1:1000, CST, USA), Claudin-5(1:1000, Invitrogen, USA), ZO-1(1:1000, Invitrogen, USA).

### Transient middle cerebral artery occlusion model in mice

Transient ischemia induced by middle cerebral artery occlusion (tMCAO) surgery was performed as previously described [64, 65]. Briefly, mice were anesthetized with 2.5 % isoflurane inhalation, and body temperature was maintained at 37 ± 0.5 °C through a thermostat-controlled heating pad. The left common carotid artery, external carotid arteries (ECA) and internal carotid artery (ICA) were isolated and ligated. A 2.0 cm length of monofilament nylon suture (size, 7–0), with its tip coated with silicon resin, was inserted from the right ECA into the lumen of ICA, then advanced until resistance was felt (0.8–1.0 cm from the bifurcation). Reperfusion was initiated by withdrawal of the monofilament after 90 min occlusion. Surgeries were finished and animals were placed back into their cages. Pain and discomfort were minimized by an initial injection of buprenorphine (0.1 mg/kg, sc) and Carperofen (5 mg/kg, sc) followed with another Carperofen injection every 24 h.

### Animal experimental design

Mice were included and randomly divided into Sham group, model group, n-EMVs group and OGD- EMVs group. In model group, mice were operated with tMCAO surgery. The sham-operated mice underwent the same procedure, except that the monofilament was inserted. The n-EMVs group or OGD- EMVs group were respectively treated with 50 μg n-EMVs or OGD- EMVs in 100 μL PBS via tail intravenous injection after 30 min of tMCAO surgery. The various groups of mice were subjected to the subsequent measurements of evans blue extravasation, cerebral blood flow, infarct volume and neurological deficits. All experiments were approved by the Laboratory Animal Care and Use Committees at Guangdong Medical University in accordance to the Guide for the Care and Use of Laboratory Animals issued by the National Institutes of Health.

### Evans blue extravasation

BBB injury of mice (*n* = 32) in each group was evaluated 48 h after tMCAO by Evans blue dye [66]. In brief, 4 % of evans blue dye (Sigma-Aldrich, StLouis, MO, USA) in 0.9 % saline (2 mL/kg) was injected into the tail vein. Three hours later, mice were sacrificed. For observation of evans blue extravation, the mouse brains were removed quikly and sliced into five 2 mm-thick coronal section. Injured brain tissue was dyed blue. For measuring the amount of extravasated evans blue, the mouse brains were homogenized in 1 mL of 50 % trichloroacetic acid and centrifuged. And then the supernatant was diluted four-fold with ethanol. A fluorescent plate reader (620 nm excitation and 680 nm emission) was used to determine dye concentrations. The amount of extravasated Evans blue was expressed as nanograms per brain.

### Immunofluorescence microscopy analysis of EMVs-astrocytes fusion on brain slices of tMCAO mice

The PKH26 labeled n-EMVs (50 μg in 100 μl PBS) were injected into mice for 24 h. After that, brains were dissected from mice and frozen in liquid nitrogen. Frozen brains were cut into 20-μm-thick sections using a cryostat. The sections were mounted on coverslips, air dried, treated with 0.2 % Triton-100 for 15 min at room temperature, and then fixed in stationary liquid at 4 °C for 30 min. After washing with scrub solution, and incubated with primary antibodies (GFAP 1:5000, abcan, USA) at 4 °C for the night, sections were incubated with secondary antibodies for 30 min. After rinsing with scrub solution, the sections were embedded on the microscope stage. Images were obtained using immunofluorescence microscope equipped with a UPlanApo × 40 objective lens.

### Measurement of cerebral blood flow

Forty-eight hours after tMCAO following EMVs or PBS transfusion, the CBF of mice (*n* = 32) from various groups was determined by the PeriCam PSI System (perimed, Sweden) as previously described [67–69]. Briefly, mouse was anesthetized with 2.5 % isoflurane and placed on a stereotaxic apparatus. A crossing skin incision was made on the head to expose the whole skull. PeriCam PSI System scanning (2.0 × 1.4 cm) was performed on the intact skull for approximately 1 min. The mean blood perfusion of the ischemic hemisphere was analyzed with the soft ware (Pimsoft).

### Measurements of infarct volume and neurological deficits

Cerebral damage was measured using 2 % 2,3,5-triphenyltetrazolium chloride (TTC) staining as described previously [70]. Briefly, the brains of mice (*n* = 32) from each group were quickly removed and sliced into five coronal sections (2 mm thick). Then the slices were stained with 2 % TTC for 15 min at 37 °C. The infarct area and total area were measured by Image J (Bethesda, MD, USA) software and the percentage of infarct area was calculated.

The 5-point scale method was used to evaluate the neurological deficit scores as we previously described [71]. The neurological deficit scores were determined by five points scale: 0, normal motor function; 1, flexion of contralateral torso and forelimb upon lifting the whole animal by the tail; 2, circling to the contralateral side but normal posture at rest; 3, leaning to the contralateral side at rest; 4, no spontaneous motor activity. The neurologic behavior of mice was scored by an investigator who was unaware of animal grouping.

### Statistical analysis

Data were expressed as mean ± SEM. Comparisons for two groups were performed by using Student’s t-test (GraphPad Prism 5 software). Multiple comparisons were performed by one-way ANOVA. *p*-values <0.05 were considered to be significant.

## Abbreviations

Akt, serine/threonine kinase; BBB, blood brain barrier; CNS, central nervous system; ECs, endothelial cells; EMVs, endothelial microvesicles; GFAP, the Glial Fibrillary Acidic Protein; HBMECs, human brain microvascular endothelial cells; ICH, intracranial hemorrhage; IS, ischemic stroke; MTT, 3-[4,5-dimethylthiazyol-2yl]-2,5-diphenyltetrazolium bromide; n-EMVs, EMVs derived from HBMECs cultured in normal condition medium; OGD- EMVs, EMVs derived from HBMECs cultured in oxygen and glucose deprivation condition; PI3K, phosphatidylinositol 3 kinase; tMCAO, transient middle cerebral artery occulsion; TTC, 2,3,5-triphenyltetrazolium chloride

## Additional files

Additional file 1: Availability of data for EMVs identification. (XLS 17 kb) Additional file 2: Availability of data for proliferation, GFAP, PI3K and Akt protein expression in vehicle, OGD-EMVs and n-EMVs treated astrocytes. (XLS 20 kb) Additional file 3: Availability of data for apoptotic rate, cleaved caspase-9 and Bcl-2 protein level in vehicle, OGD-EMVs and n-EMVs treated astrocytes. (XLS 19 kb) Additional file 4: Availability of data for FITC-Dextran Flux, claudin-5 and ZO-1 protein level in vehicle, OGD-EMVs and n-EMVs treated astrocytes. (XLS 19 kb) Additional file 5: Availability of data for Evans blue dye extravasation in tMCAO mice after treated with OGD-EMVs and n-EMVs. (XLS 18 kb) Additional file 6: Availability of data for CBF, infarct volume and neurological deficits of tMCAO mice after treated with OGD-EMVs and n-EMVs. (XLS 12 kb)
